# Endocrine Disrupting Chemicals, Hormone Receptors, and Acne Vulgaris: A Connecting Hypothesis

**DOI:** 10.3390/cells10061439

**Published:** 2021-06-09

**Authors:** Akshatha Rao, Sotonye C. Douglas, Julianne M. Hall

**Affiliations:** Frank H. Netter MD School of Medicine, Quinnipiac University, North Haven, CT 06473, USA; akshatha.rao@quinnipiac.edu (A.R.); Sotonye.Douglas@quinnipiac.edu (S.C.D.)

**Keywords:** endocrine disrupting chemicals, acne vulgaris, hormones, nuclear hormone receptors, androgens, estrogens, phytoestrogens

## Abstract

The relationship between endocrine disrupting chemicals (EDCs) and the pathogenesis of acne vulgaris has yet to be explored in the literature. Acne vulgaris is a chronic inflammatory skin disease of the pilosebaceous unit. The pathogenesis of acne involves several hormonal pathways, including androgens, insulin-like growth factor 1(IGF-1), estrogens, and corticosteroids. EDCs influence these pathways primarily through two mechanisms: altering endogenous hormone levels and interfering with hormone receptor function. This review article describes the mechanistic links between EDCs and the development of acne lesions. Highlighted is the contributory role of androgen receptor ligands, such as bisphenol A (BPA) and mono-2-ethylhexyl Phthalate (MEHP), via upregulation of lipogenic genes and resultant exacerbation of cholesterol synthesis. Additionally discussed is the protective role of phytoestrogen EDCs in counteracting androgen-induced sebocyte maturation through attenuation of PPARy transcriptional activity (i.e., resveratrol) and restoration of estrogen-regulated TGF-B expression in skin cells (i.e., genistein). Examination of the relationship between EDCs and acne vulgaris may inform adjunctive avenues of treatment such as limiting environmental exposures, and increasing low-glycemic, plant-rich foods in the diet. With a better understanding of the cumulative role that EDCs play in acne, clinicians can be better equipped to treat and ultimately improve the lives of their patients.

## 1. Introduction

Acne vulgaris is a chronic inflammatory skin disease involving the pilosebaceous unit [1]. Its global prevalence is estimated at 9.4%, ranking as the 8th most prevalent disease worldwide [2]. Acne is extremely common among adolescents, with over 85% of 12–15-year-olds affected. However, it is also increasing in prevalence among adults, particularly among women [3,4].

The psychological ramifications associated with acne can be severe and life-long. Patients with acne are at a higher risk for lower self-esteem, anxiety, depression, suicidal ideation, and even unemployment [5]. Long-term sequelae such as ‘skin picking’ and scarring further exacerbate the negative psychological effects that acne brings. Understanding the pathogenesis of acne is crucial to finding effective treatment regimens and alleviating the burden that acne brings to both adolescent and adult populations.

The role of androgens in the pathogenesis of acne has been documented and covered in many reviews. For example, Zoubalis et al. explains the many clinical observations that support its role-positive associations between serum androgen levels and acne lesions counts, the near-absence of acne in men with androgen insensitivity syndrome or early castration, and acne formation in small children with virilizing tumors or congenital adrenal hyperplasia (CAH) [6]. Interestingly, hormonal treatment of acne in female patients may involve spironolactone, a drug that suppresses androgen receptor signaling [7].

Endocrine disrupting chemicals (EDCs) are exogenous chemicals found in the environment that interfere with hormone function. They are present in many consumer items, plasticizers, pharmaceuticals, groundwater, and agricultural products. Recently EDCs have gained much spotlight due to their potential to disrupt many neurodevelopmental and endogenous sex hormone pathways, even at low levels [8,9]. Due to the ubiquitous exposure to EDCs and their ability to alter key hormone functions, we propose that EDCs may be relevant in the development of acne.

This review examines the potential contribution of EDCs to the pathogenesis of acne vulgaris. Included is the current understanding of acne pathogenesis and its relationship with hormones, and the known effects of EDCs on endogenous hormones. Evidence of human exposures to EDCs is discussed as well as potential mechanisms in which EDCs can contribute to the development of acne. We conclude with a brief discussion of therapeutics, practical suggestions for clinical practice, and avenues for future research.

## 2. Relationship between Hormones and the Pathogenesis of Acne

The pathogenesis of acne is multifactorial and includes the release of inflammatory mediators, altered keratinization, comedone formation, altered sebum production, and follicular colonization by the bacterium *Propionibacterium acnes* (*P. acnes*) [1,10,11].

### 2.1. Hormones and the Sebaceous Gland

The sebaceous gland is a key component involved in acne. Its normal function is to secrete sebum, a group of triglycerides, fatty acids, and esters that lubricate the skin, transport anti-oxidants, protect against ultra-violet (UV) rays, and promote anti-bacterial activity [10]. Excess sebum production or alteration in its fatty acid composition can interfere with follicular keratinization, leading to pore blockage and the formation of comedones. A sebum-rich environment also promotes colonization by *P. acnes*.

#### 2.1.1. Androgens

Excess sebum is attributed to the potent androgen 5α-dihydrotestosterone (5α-DHT), which is converted from testosterone via 5α-reductase type 1, an enzyme expressed mainly in the skin, particularly in facial sebocytes and sweat glands [6,10].

5α-DHT exerts its actions on the sebaceous gland via the nuclear androgen receptor (AR). [6,11]. Genetic studies have shown that dysregulation of the AR occurs in people experiencing severe acne. For example, as cited in a review by Ju et. al, a study of European Americans found a significant association between teenagers with severe acne and a Myc protooncogene related to upregulation of AR on the chromosome 8 q24 region [11]. Furthermore, in vivo topographic analysis has revealed a statistically significant increase in AR expression in the T-zones of the face, known to have higher sebum production when compared to the U-zones of the face [11]. An overall increase in sebum production in both of these zones was also found in patients with acne when compared to controls [12]. However, there was no correlation between the quantity of sebum and acne lesion count in most areas, suggesting the pathogenesis of acne is multi-faceted [12].

In vitro experiments have revealed that androgens like testosterone and 5α-DHT do not alter sebum synthesis completely on their own. They likely require in vivo co-factors such as peroxisome proliferator-activated receptors (PPAR) ligands in order to mediate their effects on the sebaceous gland [6,10]. In fact, evidence suggests a role of other PPAR ligands such as leukotrienes B4 (LTB4) in the development of inflammatory acne lesions as well [13].

#### 2.1.2. IGF-1

End organ receptor sensitivity of the AR is particularly relevant to the processes underlying acne. One hormone that has been gaining focus is insulin-like growth factor 1 (IGF-1). During puberty, there is a surge in growth hormone (GH) and subsequent IGF-1 release. IGF-1 induces adrenal and gonadal secretion of androgens, and also acts on the sebaceous gland via the GF-1/AKT/mTORC1/SREBP1 signaling pathway. End-results of this pathway include an increase in the conversion of testosterone to 5α-DHT (which has a higher affinity for AR), increased end-organ receptor sensitivity to androgens, and increased expression of PPARγ [10,14]. The relationship between IGF-1 and androgens and their effects on the sebaceous gland have brought into question whether high glycemic loads can lead to increased IGF-1 release and thus an increased risk of acne.

#### 2.1.3. Estrogens

Estrogens suppress sebaceous gland function by opposing androgens. Estrogens are also thought to be implicated in wound-healing and anti-inflammatory processes through a complex interaction with IGF-1 [11,14]. A systematic review screening over 1000 studies found that patients with acne vulgaris had lower serum estrogen levels when compared to controls [15]. This suggests that estrogen may be another hormone involved in the pathogenesis of acne.

#### 2.1.4. CRH and Cortisol

Corticotrophin-releasing hormone (CRH) and cortisol are stress-linked hormones that also mediate sebaceous activity. Very strong expression of CRH has been observed in the sebaceous glands of acne-involved skin when compared to unaffected skin [16,17]. CRH inhibits proliferation of sebocytes, stimulates sebum production, and increases expression of the enzyme Δ5-3β-hydroxysteroid dehydrogenase, which activates androgens [16].

CRH and cortisol are considered stress hormones because they are released in the body during times of psychological stress. Several studies have indicated a significant association between stress levels and acne severity [18,19]. It is possible that the increase in CRH and cortisol during stressful periods contributes to the development of acne lesions through its mechanisms on the sebaceous gland, as previously described. However, studies have not looked at this directly. In fact, although one study of 94 adolescent students in Singapore found a significant relationship between stress levels during examinations and acne severity, there was no significant difference in sebum measurements between the high-stress and low-stress conditions [18]. This suggests that mechanisms other than an increase of sebum activity contribute to the development of acne lesions in the context of stress.

#### 2.1.5. Cytokines and Inflammation

In the past, it was thought that inflammation only played a role in the later stages of acne lesions (i.e., papules, pustules, cysts). However histological and immunological evidence demonstrate that sub-clinical inflammation is present even throughout earlier stages of acne. Evidence of this includes upregulation of inflammatory mediators (IL-1, CD3+, CD4+, macrophages) in uninvolved skin and IL-1 α activity in open, formerly considered “non-inflammatory”, comedones [20]. We now understand that these inflammatory processes likely precede the hyper-keratinization process in acne, confirming that acne is ultimately considered a chronic, inflammatory disease.

Inflammatory mediators are thought to be activated by several factors including the bacterium *P. acnes* activating toll-like receptor 2 (TLR2), altered sebum composition, and disruption of the oxidant/antioxidant ratio in skin surface lipids [10,20]. Evidence has shown that *P. acnes* is not necessarily required for the development of inflammation in acne, signifying that sebaceous glands hold an important role in inciting inflammatory events in acne.

Modifications in the lipid composition of sebum are thought to trigger the formation of acne lesions. Lipoperoxides, particularly those from the degradation of squalene, appear to be a culprit. One study found that lipoperoxides exist in significantly higher concentrations in the comedones of patients with acne when compared to healthy skin [21]. Lipoperoxides affect keratinocyte proliferation and stimulate the release of pro-inflammatory cytokines [20,22]. They are also ligands of PPARy [10], a nuclear hormone receptor, which as mentioned above, facilitates the effects of androgens on acne progression.

A cytokine mRNA analysis revealed that facial acne lesions had evidence of significantly greater levels of tumor necrosis factor-α (TNF- α) and interleukins [23]. Levels of IL-8 in particular showed a 3000-fold difference in acne lesions when compared to the un-involved skin. Interestingly, another study found that DHT is able to upregulate TNF- α and IL-6 in primary sebocyte cultures of the scalp [24]. This suggests that androgens not only influence sebogenesis but also have direct inflammatory effects.

## 3. Evidence of Human Exposures to EDCs

### 3.1. Classes of EDCs

Approximately 1000 diverse agents have been characterized as EDCs. A comprehensive list compiled over the past 16 years can be found at The Endocrine Disruptor Exchange (TEDX; http://endocrinedisruption.org; accessed on 4 March 2021) and is available until 2022. A consensus on the key characteristics on classification of an exogenous agent as an EDCs was recently reported [25], as discussed above (see ‘Effect of ECCs on Endogenous Hormone Pathways’).

In general, the most well-studied EDCs can be divided into 5 broad classes: plant-derived (phytoestrogens), industrial chemicals, manufactured household and consumables, medical items, and pharmaceuticals (Table 1).

### 3.2. Routes of Exposure

Numerous routes of human exposure to EDCs have been identified. These include ingestion of food, dust, and water, ingestion of breast milk, inhalation of gases and particles in the air, skin contact, intravenous tubing, or biological transfer across the placenta [34].

### 3.3. Evidence of Exposure

There is concrete evidence of human exposure to EDCs. Detectable levels of EDCs exist in numerous biological fluids of both children and adults, including blood, urine, sweat, breast milk, and hair [35,36,37]. Since 1960, the Center for Disease Control (CDC) has been engaged in an active surveillance program of US residents, termed the National Health and Nutrition Examination Survey. In the past 20 years, NHANES studies have revealed that greater than 95% of the representative population test positive for numerous EDCs including phthalates, BPA, and pesticides. As part of the 2013–2014 NHANES, detectable levels of BPA were found in 96% of urine samples taken from approximately 2500 individuals 6 years and older [38], and 95–98% of women and children tested positive for phthalates in two NHANES surveys conducted from 1999 to 2014 [39].

Despite the knowledge that EDCs are readily detectable in humans, whether they accumulate at sufficient levels to become physiologically disruptive has been long debated. Experimental studies confirm that indeed, EDCs elicit adverse effects in many systems, however, it is unclear whether these findings can be extrapolated to human exposure levels when considering the relatively high doses required to elicit such responses. However, an emerging wealth of recent studies have documented adverse effects of EDC exposure at low doses [40,41,42], suggesting that existing EDC levels in human body fluids are sufficient to exert substantial physiological consequences. It is known that the lipophilic nature of many EDCs permits bioaccumulation in human tissues (e.g., adipose) or body fluids to substantial levels [43]. For example, following feeding, infants consuming exclusively soy-based formula had serum genistein levels of 1–10 μM [44], which are certainly within the range to elicit physiological effects through estrogen receptors. There is also evidence that different classes of EDCs can exert additive or even synergistic effects [45], and given that many environmental pollutants are found in mixtures, concurrent contact with numerous agents may provide an additive effect.

## 4. Effects of EDCs on Endogenous Hormone Pathways

EDCs have various effects on endogenous hormone pathways, particularly those of hormones relevant to the pathogenesis of acne: androgen, estrogen, and cortisol. Two predominant mechanisms discussed below involve (I.) alterations in hormone levels and (II.) interference with hormone receptor function.

### 4.1. EDC-Mediated Alteration of Endogenous Hormone Levels

#### 4.1.1. Androgens

It has recently been proposed that EDCs may promote the pathogenesis of acne by raising endogenous androgen levels [46]. A wide-array of EDCs, including BPA and phthalates, are known to possess androgenic activities, and NHANES studies indicate that the majority of humans (greater than 95%) possess detectable serum and urinary levels of these agents [38,39]. Furthermore, a recent study showed that the levels of BPA and phthalates were significantly elevated in adolescent girls with PCOS [47]. Furthermore, BPA is thought to increase androgen levels in females by inhibiting aromatase activity in ovarian granulosa cells [48].

#### 4.1.2. Estrogens and IGF-1

Estrogens have protective actions in acne that may indeed be disrupted by the ability of EDCs to alter endogenous hormone levels. In granulosa cells from human IVF patients, BPA was shown to decrease proliferation and FSH-induced aromatase expression, thus potentially altering the balance to favor androgen biosynthesis. The proposed mechanism involved BPA-mediated upregulation and activation of PPARy and increased expression of IGF-I and its receptor [43]. As discussed above, IGF-1 increases androgen sensitivity and activates sebaceous gland activity; thus, it is possible that BPA may exacerbate acne by enhancing the ratio of androgen-to-estrogen production and facilitating androgen sensitivity via IGF-1 signaling.

Exposure to numerous other EDCs has been associated with a decline in estradiol biosynthesis. Phthalates and PCBs are known to decrease several steroidogenic enzymes required for estradiol biosynthesis, including aromatase [8], potentially altering the estradiol-to androgen ration as discussed above as related to acne.

#### 4.1.3. CRH and Cortisol

CRH and cortisol enhance sebaceous gland activity and therefore are key players in the pathogenesis of acne. Experimental studies in rodents showed that prenatal exposure to PCBs resulted in elevated cortisol levels in female animals [49]. In humans, inhalation of the essential oil, Clary sage, was associated with a decline in cortisol levels in postmenopausal women [50]. Thus, while EDCs may alter endogenous cortisol levels, the link between cortisol, cutaneous CRH expression, and activity is not yet understood.

## 5. EDC-Mediated Interference with Hormone Receptor Function

### 5.1. EDCs, Hormones and their Receptors

The most established effects of EDCs on endogenous hormone pathways involve interactions with nuclear hormone receptors (NHR), particularly as it relates to acne, the receptors for estrogens (ERs), androgen (ARs), cortisol/glucocorticoids (GR), and prostaglandins (PPARy). According to the classical pathway of NHR action, NHRs are ligand-activated transcription factors that reside in the cytoplasm or nucleus in the quiescent state. Upon binding hormones, NHRs undergo conformational changes that enable relocation of receptors to the chromatin-containing regions of the nucleus and subsequent association with transcriptional coregulators. Receptor–coregulator complexes bind DNA at specific recognition sequences in the regulatory region of target genes, enabling interactions with the general transcriptional apparatus to alter (induce or repress) target gene expression. NHR coregulators possess chromatin remodeling activities, and thus, association of receptor–coregulator complexes may accelerate or hinder the access and activity of the general transcriptional apparatus at transcription start sites. NHR-mediated changes in gene expression and cellular protein content mediate essential developmental and homeostatic processes [51,52].

### 5.2. Molecular Mechanisms of EDC Action on Nuclear Hormone Receptors

#### 5.2.1. Effects on EDCs on Classical Nuclear Hormone Receptor Pathways

EDCs have been shown to interfere with all of these aspects of classical NHR action, as discussed below.

#### 5.2.2. Ligand Binding

EDCs interact with the ligand-binding pockets of numerous NHRs, including ERs, ARs, PPARy [53], and GRs [54].

#### 5.2.3. Agonist/Antagonist Activity of EDCs

A key paradigm of NHR action is that different ligands induce unique conformational changes in NHRs, resulting in distinct biological activities [55,56]. EDCs are known to induce diverse conformational changes in NHRs that enable these agents to possess a wide range of different activities: NHR agonist, partial agonist, antagonist, or mixed agonist/antagonist [53,54].

#### 5.2.4. Recruitment of Transcriptional Coregulators

Ligand binding results in conformational changes within NHRs that exposes surfaces for interaction with transcriptional coregulators. The differential agonist/antagonist activities of various NHR ligands are thought to, in part, reflect unique ligand-specific coregulator recruitment [57,58]. Evidence suggests that this is also true for EDCs, as EDC-bound ERs were shown to associate with transcriptional coactivators in a manner that differed from estradiol or other ER ligands [59,60]. EDC-specific coregulators have not been identified, nor have those for specific various natural and synthetic ligands for NHRs. Rather, it is believed that the agonist/antagonist activity of specific natural, synthetic, or EDC ligands on a particular NHR reflects in part differential engagement of physiological coregulators that are used by diverse ligands.

#### 5.2.5. DNA Binding and Gene Expression

DNA is an allosteric modulator of NHR function that, together with ligand, permits transcriptional coregulator recruitment and target gene specificity [28,60]. Like endogenous NHR ligands, EDCs display differing activities on distinct target genes [28]. Notably, EDCs are known to selectively enhance AR and PPARy-mediated expression of lipogenic genes and subsequent lipogenesis [61,62], providing a plausible link between EDCs exposure and enhanced sebaceous gland activity in acne.

#### 5.2.6. EDCs and Nongenomic NHR Signaling

In addition to their genomic functions, some NHR ligands mediate nongenomic signaling through membrane-associated hormone receptors. Nongenomic signaling by estrogens is the most well-studied of nuclear hormones. Membrane-initiated estrogen pathways are mediated through a cell-surface receptor termed G protein-coupled estrogen receptor 1 (GPER1) that displays specificity for estradiol compared to steroid hormones [63]. Estradiol binding of GPER1 activates adenylyl cyclase and release of membrane-tethered epidermal growth factor (EGF), culminating in a series of rapid intracellular events [63,64]. In experimental studies, genetic deletion of GPER30 was associated with glucose intolerance, insulin resistance, obesity, and cardiovascular pathology, highlighting the crucial role of this estradiol signaling pathway in numerous aspects of homeostasis [65].

Numerous EDCs bind GPER30 with a similar affinity to estradiol and elicit estrogen-like intracellular responses [26,66]. For example, BPA has been shown to augment insulin release from pancreatic beta cells via activation of nongenomic ER signaling [27]. In regards to acne, this pathway has potential to exacerbate sebocyte activity and inflammation via insulin-mediated elevation of androgen and IGF-1 activities.

#### 5.2.7. Epigenetic Effects of EDCS

Exposure to EDCs during critical developmental periods can result in permanent changes in the programming of normal biological processes in a manner that may enhance one’s susceptibility to diseases in adulthood. Termed ‘developmental reprogramming,’ these events are a type of gene–environment interaction during the pre- and peri-natal periods that involve epigenetic modifications made on the genome. Epigenetic modifications include methylation of histones, and methylation of cytosine and guanine-rich stretches of DNA (CpG islands), which are changes that may alter gene expression [25]. Although methylation is an essential aspect of normal development, inappropriate exposure to exogenous stressors during key developmental periods may predispose to cancer in adulthood as a consequence of dysregulated epigenetic modifications [29]. A number of EDCs with ubiquitous exposure among the general population are known to induce epigenetic reprogramming [25]. Indeed, recent evidence suggests that these effects may be contributory to the rise of obesity and insulin resistance in the general population [30,31]. A recent study showed that EDC exposure led to altered gene methylation and increased functional activity of PPARy [32]. Given the link between PPARy activity and pathogenesis of acne, the relationship between epigenetic modifications and acne warrants great study. Indeed, there is emerging evidence that some of the therapeutic effects of retinoids in acne may be attributed to epigenetic changes that favor suppression of lipogenesis and upregulation of skin remodeling pathways [33].

## 6. Potential Mechanistic Links Between EDC Exposure and Acne

As numerous hormones play a critical role in the pathogenesis of acne, below, the hypothesis that exposure to these agents may play a causative or protective role in the etiology of acne is examined.

### 6.1. Androgens

Many topical formulations contain mixtures of EDCs, including tea tree and lavender oils, which are popular choices for treating acne. Tea tree and lavender oils contain several chemicals that together and alone display anti-androgenic activities in skin [67,68], which may lend to their therapeutic effects in acne. Other evidence of potential protective effects of EDCs in acne was seen in clinical trials of women with PCOS. Daily treatment of the phytoestrogen resveratrol for 3 months caused a 23% reduction in circulating testosterone. Although the authors did not use acne as an endpoint, they did note that resveratrol consumption was associated with decline in other clinical symptoms associated with testosterone excess [69].

There is evidence that EDC exposure may facilitate the process of androgen-induced sebocyte maturation. In sebaceous glands, androgens increase sebum production via upregulation of numerous lipogenic genes including sterol regulatory element binding proteins (SREBPs), acetyl-CoA carboxylase-1 (*ACC1*), and fatty acid synthase (FASN) [70,71]. Mono-2-ethylhexyl Phthalate (MEHP), a metabolite of a widely used industrial plasticizer, was shown to promote lipogenesis and expression of SREBP-1c, ACC, FASN, and other known AR-regulated genes [61]. BPA, which like MEHP, is a AR ligand, was shown to exacerbate cholesterol synthesis through regulation of AR target genes including 3-Hydroxy-3-methylglutaryl coenzyme A reductase (HMGCR) and SREBP-2 [72].

An additional intersection of EDCs and androgens in acne may exist in the inflammatory aspect of acne. Genistein, an established androgen receptor ligand and modulator [73], was shown to inhibit activation and subsequent inflammatory cytokine production by mammalian macrophages [74]. Collectively, the studies discussed above provide evidence that phytoestrogens may possess the potential to exacerbate or confer protection in acne by interaction with androgen signaling pathways.

### 6.2. Estrogens

**Figure 1:**
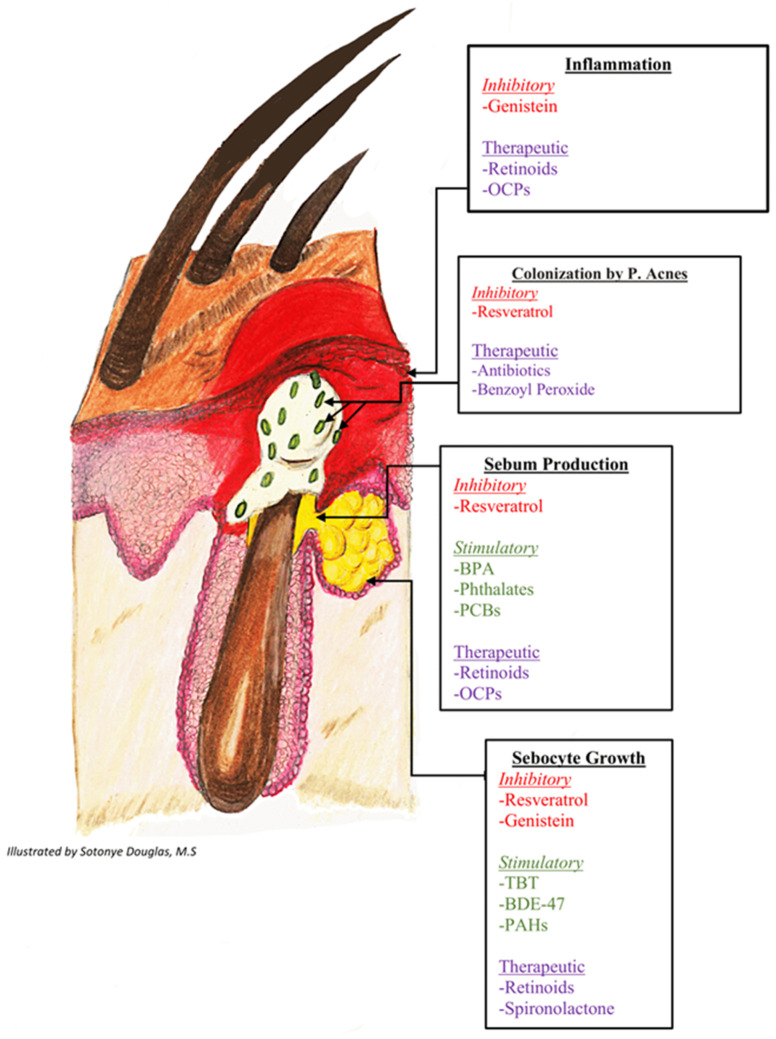
Stimulatory and inhibitory effects of endocrine disrupting chemicals on pathways of acne pathogenesis. ^1^ Genistein’s inhibitory effects are mediated by restoration of TGF-B expression and inhibition of inflammatory cytokine production. Resveratrol’s inhibitory effects are driven by downregulation of PPARy expression and inhibition of lipogenesis. ^2^ BPA’s stimulatory effects are driven by exacerbated cholesterol synthesis through regulation of androgen receptor target genes and direct stimulation of the glucocorticoid receptor. ^3^ Phthalates and PCBs’ stimulatory effects occur via decreased aromatase activity. ^4^ TBT, BDE-47, and PAHs’ stimulatory effects occur via altered methylation of PPARy target genes. TGF-B: Transforming growth factor-beta; BPA: bisphenol A; PPARy: peroxisome proliferator-activated receptor gamma; IGF-1: insulin-like growth factor-1; PCBs: polychlorinated biphenyls; TBT: Tributyltin; BDE-47: polybrominated diphenyl ether 47; PAHs: polycyclic aromatic hydrocarbons.

The observation that dietary intake of soy products is associated with lower incidence of acne [75] is consistent with the known protective role of estrogens in the etiology of acne. A potential mechanistic link between soy consumption and acne may in part involve transforming growth factor beta (TGFβ), a cytokine that is thought to be estrogen-ER regulated [76]. In skin, TGFβ plays a critical role in sebaceous glands by maintaining sebocytes in an undifferentiated state [77]. Genistein was shown to restore TGFβ expression in skin of ovariectomized animals [78]. Thus, the ability of genistein to replace estrogen signaling in the skin may contribute to its postulated therapeutic effects in acne, particularly reductions in inflammation and sebocyte growth (Figure 1).

### 6.3. CRH and Cortisol

There is emerging evidence that EDCs may interact with the cortisol pathway via direct stimulation of the glucocorticoid receptor (GR). Several EDCs, including BPA and phthalate, were shown to activate GR and promote GR-dependent lipogenesis and adipogenesis [79]. Given the link between CRH, cortisol, and sebum production, therefore, it is plausible that some EDCs may exacerbate acne via activation of GR-dependent lipogenesis in sebocytes.

### 6.4. PPARy

Increased sebum production has been noted in patients receiving PPARy agonists (thiazolidediones) for type 2 diabetes, suggesting that activation of the receptor at clinically-relevant doses is sufficient to exacerbate acne [80]. Furthermore, some EDCs, such as tibutylin (TBT), polybrominated diphenyl ether 47 (BDE-47), and polycyclic aromatic carbons (PAHs), have been shown to alter methylated and increase the functional activity of PPAR expression, suggesting that these chemicals are relevant in acne [32].

Resveratrol has recently received attention for its potential therapeutic effects in acne. Resveratrol was found to inhibit sebocyte growth in part via downregulation of PPARy expression and associated lipogenesis [81]. Recent studies have defined the potential cellular and molecular mechanisms by which resveratrol may modulate PPARy-dependent lipogenesis to achieve therapeutic effects in acne. In a mammalian fibroblast system of adipocyte differentiation, resveratrol was shown to inhibit lipogenesis and other key features of adipocyte maturation. These effects were mediated via resveratrol-dependent attenuation of PPARy transcriptional activity and receptor association with transcriptional coregulators. Coincident analysis of gene expression revealed a resveratrol-mediated downregulation of a series of PPARy target genes involved in lipid metabolism, including fatty acid binding protein and phosphoenolpyruvate carboxykinase [82].

## 7. EDCs and Acne Therapeutics

The treatment of acne has been previously reviewed [1,7,83,84,85,86]. Treatment goals include clearance of lesions, maintenance of clearance, and minimization of potential sequelae such as PIH, scarring, and erythema. Common agents used include retinoids, antibiotics, benzoyl peroxide, oral contraceptives, and androgen blockers. These therapeutics target different mechanisms involved in the development of acne lesions. Not previously described are potential areas of interaction between EDCs, retinoids, and hormones, as is briefly examined below.

### 7.1. EDCs and Retinoids

Retinoids are vitamin A-derived agonists of the retinoic acid receptors α, β, and γ (RAR- α, RAR-β, RAR-γ) [86,87]. Retinoic acid receptors are a group of nuclear hormone receptors that form into a homo/hetero-dimer upon ligand binding. This dimer complex then binds to nuclear responsive elements RARE (retinoic acid response element) or RXRE (retinoid X receptor response element) to activate transcription of gene expression [87]. As previously discussed, EDCs are known to participate in all aspects of nuclear hormone receptor function (See “Effects of EDCs on Endogenous Hormone Pathways”). If certain EDCs act as an agonist, antagonist, or recruiter of coregulators for the retinoic acid receptors, they could potentially augment or interfere with acne treatment. The efficacy of topical retinoids is dose-dependent, further posing the question of whether there are exogenous agents interfering with therapeutic responses [88].

### 7.2. EDCs and Hormonal Therapies

Combined oral contraceptives (COCs) containing estrogen are recommended as a second-line treatment of acne in females due to their anti-androgenic and anti-inflammatory properties. COCs decrease androgen production, block the AR, reduce 5-alpha reductase activity, and increase levels of sex-hormone binding globulin (SHBG)—all of which decrease circulating levels of androgens [86]. Spironolactone, an AR antagonist, is also sometimes used off-label and is effective in adult female patients with acne and adolescents with PCOS [7]. As previously discussed, there is evidence that numerous EDCs interact with several hormonal pathways, including androgens, estrogens, IGF-1, and CRH/cortisol (See “Effects of EDCs on endogenous hormone pathways”). These same pathways are targeted by oral contraceptives and androgen antagonists. This is important to consider when discussing treatment of acne because there could be direct antagonizing effects between androgenic EDCs and Spironolactone, for example, potentially interfering with treatment success.

## 8. Discussion

The persistent industrialization of society during the past century has resulted in the increased exposure of humans to a wide variety of exogenous chemicals. As noted, many of these agents possess endocrine disrupting activities and bioaccumulate at sufficient levels in humans to alter endogenous hormone signaling. A wealth of experimental, clinical, and epidemiological studies has linked EDC exposure to the pathogenesis of cancer, reproductive disorders, cardiovascular and pulmonary disease, metabolic syndrome, and other malignancies. Thus, risk assessment of EDC exposure, particularly during critical developmental periods, is paramount to reducing the disease burden.

The role of hormones in the pathogenesis of acne vulgaris is well established, and in this review, the relationship between EDC exposure and acne was examined. Indeed, as described, EDCs interfere with several key endogenous hormonal pathways that are common to acne, including those mediated by androgens, estrogens, IGF-1, and CRH/cortisol. A key point of intersection exists between the hormonal and cytokine-induced lipogenic and inflammatory mediators involved in acne and seen as a consequence of EDC exposure. The interaction of EDCs with hormonal acne therapeutics is also likely, and thus, it will be important to examine whether persistent EDC exposure may alter the efficacy of androgen and retinoid-based therapies in acne patients.

Examination of the relationship between EDCs and acne vulgaris may also inform about new avenues for therapy. The observation that phytoestrogens (genistein and resveratrol) are able to antagonize the lipogenic and sebocyte growth-promoting actions of androgens and PPARy suggests that plant-rich diets may benefit acne patients and have potential as part of disease management. Many studies have described the mitigation of inflammatory acne with a low glycemic, plant-rich diet [89,90,91,92], and it has been proposed that the therapeutic effects of diet were mediated via alterations in PPARy, IGF-1, and androgen-signaling [90].

Given the evidence demonstrating the intersection between the hormone signaling pathways of EDCs and acne, there is sufficient cause for concern to warrant limiting exposure of individuals with acne to these agents. With the increasing evidence that even low dose human exposure can be harmful, methods that can minimize topical and systemic exposure to agents known to interact with estrogen, androgen, and cortisol signaling should be considered in plans for acne management. Communication between clinicians and acne patients is critical and may include several methods to both prevent exposure and promote lifestyle changes. Below are specific recommendations for physicians to aid in their counseling of patients in ways to avoid EDCs in daily life.

- BPA exposure can be reduced with the use of products labeled BPA free, and by replacing plastics with glassware for food storage and cooking.
- Phthalate consumption can be minimized with by avoiding plastics in food prep and storage, consumption of filtered water, and the use of fragrance-free cleaning products and cosmetics.
- Exposure to hormones in dairy products can be minimized by the consumption of organic products. Likewise, pesticide consumption can be avoided with organic produce.
- Mineral sunscreens containing zinc oxide or titanium dioxide as active ingredients are much safer alternatives to those containing oxybenzone (Benzophenone-3). Oxybenzone is an established EDC with estrogenic and androgenic activities, and the agent is prevalent in the body fluids of humans [93].
- Minimize exposure to air-born EDCs (Dioxins) with smoking cessation and by wearing facial masks with air filters when outside on poor air quality days or in cities with high levels of air pollution: https://www.iqair.com/us/world-air-quality-ranking; accessed on 5 May 2021.
- Topical products containing tea tree and lavender oils should be used with caution in acne patients given that these agents also possess estrogenic and androgenic properties.
- Based on existing evidence, acne patients may be counseled to follow a low glycemic, plant-rich diet.

## 9. Future Areas of Research

The relationship between EDC exposure and acne pathogenesis can be further established by examining EDC exposure specifically in patients with acne vulgaris. Further genomic studies exploring the genes that contribute to acne progression and their intersection with EDC pathways could further define the molecular mechanisms. It will be important to evaluate hormonal acne therapeutics efficacy in relationship to EDCs in order to best counsel acne patients about minimizing exposures. Finally, humans are often exposed to mixtures of EDCs which may have additive or synergistic effects, and the relationship between multi-chemical contact and acne vulgaris remains to be determined. Regardless, it is anticipated that the connection between acne vulgaris and EDCs established in this review will raise awareness and prompt further clinical studies and inform potential preventative measures.

## Figures and Tables

**Table 1:** Classes of common EDCs.

Class	Sources	Examples	Comments
Phytoestrogens	Breads, cereals, nuts, soy, legumes, fruits, vegetables	○ Genistein (found in soy) ○ Resveratrol (enriched in grapes and tomatoes)	○ Genistein and resveratrol are known to mimic or antagonize effects of estrogens and androgens. ○ Phytoestrogens interact with a number of other hormone signaling pathways as well [26,27]
Industrial Chemicals	Pesticides, flame retardants, combustion products	○ Dioxin (toxic byproduct of numerous manufacturing processes) ○ Dichlorodiphenyltrichloroethane (DDT)	○ DDT was banned worldwide in 2001 but still persists in the environment [28]
Household and Consumer Items	Food and beverage packaging materials, contaminated foods, well-water, toys, cosmetics, sunscreens, other topical formulations	○ Phthalates ○ bisphenol A (BPA) ○ Bisphenol F (BPF) ○ Bisphenol S (BPS)	○ Phthalates, which interfere with estrogen and androgen signaling, are ubiquitous in household and consumable items. ○ Due to emerging safety concerns, BPA was banned from use in baby bottles in 2012, but manufacturers continue the use of BPA in numerous plastics. ○ In some instances, BPA is starting to be replaced by Bisphenol F (BPF) and Bisphenol S (BPS), however, there is a wealth of recent evidence that these compounds BPS and BPF are as hormonally active as BPA, with similar estrogenic, anti-estrogenic, androgen, and anti-androgenic activities [29,30]
Medical Devices	disposable gloves, plastic devices, intravenous tubing	○ Bisphenol A	○ BPA and phthalates are the most widespread EDCs present in healthcare and medical devices [31]
Pharmaceuticals	Leakage into drinking water and soil	○ Natural or synthetic steroids (i.e., estrogens) ○ Diethylstilbestrol (DES)	○ Both DES and natural estrogens are detectable and present at sufficient concentrations to manifest biological effects as demonstrated in animals and postulated in humans [32,33].
